# Reference gene identification for normalisation of RT‐qPCR analysis in plasma samples of the rat middle cerebral artery occlusion model

**DOI:** 10.1002/vms3.879

**Published:** 2022-07-27

**Authors:** Hui Zhou, Xin Yang, Jiayi Yu, Jingyi Xu, Ruiwen Zhang, Ting Zhang, Xijie Wang, Jing Ma

**Affiliations:** ^1^ Shanghai Innostar Bio‐tech Co. Ltd. China State Institute of Pharmaceutical Industry Shanghai People's Republic of China

**Keywords:** circulating miRNA, middle cerebral artery occlusion, reference gene, RT‐qPCR

## Abstract

**Objective:**

In quantitative reverse transcription‐polymerase chain reaction (RT‐qPCR) studies, the selection and validation of reference genes are crucial for the accurate analysis of MicroRNAs (miRNAs) expression. In this work, the optimal reference genes for RT‐qPCR normalisation in plasma samples of rat middle cerebral artery occlusion (MCAO) models were identified.

**Methods:**

Six rat MCAO models were established. Blood samples were collected before modelling and approximately 16–24 h after modelling. Two commonly used reference genes (*U6* and *5S*) and three miRNAs (*miR‐24*, *miR‐122* and *miR‐9a*) were selected as candidate reference genes, and the expression of these genes was detected with RT‐qPCR. The acquired data were analysed using geNorm, Normfinder, BestKeeper, RefFinder and comparative delta threshold cycle statistical models.

**Results:**

The analysed results consistently showed that *miR‐24* was the most stably expressed reference gene. The ‘optimal combination’ calculated by geNorm was *miR‐24*, *U6* and*5S*. The expression level of the target gene *miR124* was similar when the most stable reference gene *miR‐24* or the ‘optimal combination’ was used as a reference gene. However, compared with *miR24* or the ‘optimal combination’, the less stable reference genes influenced the fold change and the data accuracy with a large standard deviation.

**Conclusion:**

These results confirmed the importance of selecting suitable reference genes for normalisation to obtain reliable results in RT‐qPCR studies and demonstrated that the identified reference gene *miR‐24* or the ‘optimal combination’ could be used as an internal control for gene expression analysis in the rat MCAO model.

## Graphical abstract

This study aimed to identify the optimal reference genes for RT‐qPCR normalization in plasma samples of rat Middle Cerebral Artery Occlusion (MCAO) models. MCAO models can mimic the cerebral ischemic/reperfusion injury, therefore, the present study is crucial for successful biomarker discovery and validation of the diagnosis of brain ischemia.

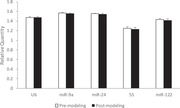

## 1 INTRODUCTION

MicroRNAs (miRNAs) are small non‐coding RNAs consisting of 19–30 nucleotides, and miRNAs regulate gene expression and play critical roles in many biological and pathological processes (Bartel, 2009). *miRNA‐124* (*miR‐124*) is almost a central nervous system‐specific miRNA that is preferentially expressed in the cerebrum and cerebellum and has been reported to be capable of protecting neurons in cerebral ischemic/reperfusion injury by regulating key processes, such as neuroinflammation (Hamzei Taj et al., 2016), oxidative stress (Kanagaraj et al., 2014) and neuronal excitability (Wang et al., 2016). The expression of *miR‐124* in peripheral blood has been reported to be influenced by cerebral ischemia and reperfusion (I/R; Sun et al., 2019). Therefore, the concentration of brain‐specific *miR‐124* in peripheral blood is a promising biomarker for I/R.

Quantitative reverse transcription‐polymerase chain reaction (RT‐qPCR) is a widely used method for quantitative analysis of circulating miRNAs in biomedical research. In the RT‐qPCR analysis, a standard curve can be constructed to calculate the absolute quantification of a particular transcript gene, or an internal reference gene can be used to calibrate and standardise a target gene in order to acquire relative quantification of the target gene and avoid the effects of differences in RNA quality, reverse transcription efficiency and PCR conditions of various samples (Brunner et al., 2004; Gutierrez et al., 2008). Ideally, the expression of reference genes should not be affected by experimental conditions or different tissues. There is no reference gene suitable for all study systems (Liu, 2005). For example, *let‐7a* and *miR‐16* are stably expressed in healthy and breast cancer tissues, but they are not suitable for the quantitative correction of miRNAs in lung cancer patients (Davoren et al., 2008; Peltier & Latham, 2008). In young rats with pentylenetetrazole‐induced seizures, the stability of nine tested reference genes, *Actb, Gapdh, B2m, Rpl13a, Sdha, Ppia, Hprt1, Pgk1 and Ywhaz*, varied significantly between different brain regions (Schwarz et al., 2020). Therefore, the stability of reference genes varies with different experimental settings, tissue types and even different tissue regions (Chapman & Waldenström, 2015). To ensure the efficiency of PCR, the selection of correspondingly stable reference genes according to different experimental conditions is an important prerequisite (Lin et al., 2014).

In the present study, we aimed to evaluate the expression of a panel of candidate reference genes in rats performed with middle cerebral artery occlusion (MCAO) operation to mimic ischemic/reperfusion injury. We selected five candidate genes (*U6 snRNA, 5S rRNA, rno‐miR‐24, rno‐miR‐122 and miR‐9a‐5p*) for identification. The five most popular algorithms, geNorm, BestKeeper, NormFinder, RefFinder and the comparative delta threshold cycle (ΔCt) approach, were used to evaluate the expression stability of the reference genes.

## 2 MATERIALS AND METHODS

### 2.1 Animals

Male Sprague‐Dawley rats at the age of approximately 6–10 weeks were purchased from the Beijing Vital River Laboratory Animal Technology Co. Ltd. The animals were group‐housed in a controlled environment with a temperature of 20–26°C and a humidity of 40%–70%. The housing room was supplied with ≥15 air exchanges per hour, with 100% fresh air (no air recirculation), and on a 12‐h light/dark cycle. Food and water were supplied ad libitum. After a 3‐day quarantine and a 2‐day acclimation, the rats were screened for the establishment of MCAO models. The study protocol was approved by the Institutional Animal Care and Use Committee of Shanghai InnoStar Bio‐Tech Co. Ltd. (IACUC No.: IACUC‐2020‐r‐078), and this study was performed in accordance with the standard ethical guidelines established by the institution.

### 2.2 MCAO model establishment

The rat MCAO model was established using the Zea‐Longa line plug method (Sempere et al., 2004). The rats were anaesthetised with 3% pentobarbital sodium at 30–45 mg/kg via intraperitoneal injection. After anaesthesia, the rats recovered on an electric blanket (kept at 37 ± 0.5°C) of the operating table in a supine position. In summary, the right common carotid artery was isolated and ligated at a distance of 1–1.5 cm from the bifurcation of the internal carotid artery and external carotid artery. A small incision of 0.2 mm was made near the head end of the common carotid artery ligation. The thread plug was inserted into the common carotid artery from the small incision and entered the internal carotid artery through the bifurcation of the internal carotid artery and external carotid artery. The thread plug was pushed along the internal carotid artery in the direction of cranial entry and inserted into the bifurcation of the anterior cerebral artery and middle cerebral artery, which blocked the blood supply of the ipsilateral internal carotid artery and contralateral internal carotid artery through the anterior cerebral artery. The time of cerebral ischemia was recorded from the successful insertion of the suture. After the operation, infrared light was used to keep the plate warm. After 2 h of cerebral ischemia, the suture left in vitro was gently pulled out for 1 cm to achieve middle cerebral artery reperfusion. The animals were housed separately after the MCAO operation.

### 2.3 Neurological score

Neurological scoring tests were carried out using Longa's scoring method (Morris et al., 2016). Zero (0) point: normal without neurological deficit; 1 point: mild focal neurologic deficit, that is, the left forepaw could not be extended when suspended by lifting the tail; 2 points: moderate focal neurological deficit, that is, turning left during crawling forward; 3 points: severe focal neurological deficit, that is, dumping to the left; 4 points, coma or cannot crawl spontaneously. The rats were tested at approximately 16–24 h post‐operation.

### 2.4 Cerebral infarction determined by 2,3,5‐triphenyl tetrazolium chloride (TTC) staining

Two rats were randomly selected after modelling. The rats were anaesthetised after the neurological scoring test, and the brain tissues were separated. The brain tissue was sliced coronally. Each slice was about 2–3 mm thick and stained with 2% TTC solution in a dark water bath at 37°C for 40 min. After staining, the brain slices were placed in 4% formalin at 4°C for 24 h. The ischemic region should be white, while the normal region should be red.

### 2.5 Extraction of total RNA

Total RNA was extracted from the plasma collected before and after MCAO model establishment using a combination of TRIzol LS reagent (Invitrogen) and the miRNeasy Serum/Plasma Kit (Qiagen) according to the manufacturer's instructions.

We used a NanoDrop 1000 to measure the quantity of the extracted total RNA at 260 nm. The samples with OD260/OD280 values of 1.8–2.0 and measured concentrations of 8–12 ng/μl were selected.

### 2.6 Synthesis of cDNA and RT‐qPCR

Using a 20‐μl reverse transcription system (miScript Reverse Transcription Kit, Qiagen), first‐strand cDNA was synthesised. Specifically, 9‐μl template RNA was combined with 4 μl 5×miScript HiFlex Buffer, 2 μl 10×miScript Nucleics Mix, 2 μl miScript Reverse Transcriptase Mix and RNase‐free water to a final reaction volume of 20 μl. Reactions were incubated for 60 min at 37°C and 5 min at 95°C. cDNA was stored at −20°C until further use.

*U6 snRNA, 5S rRNA, rno‐miR‐24, rno‐miR‐122* and *miR‐9a‐5p* were selected as reference genes for miRNA expression normalisation. RT‐qPCR was performed using the miScript SYBR Green PCR Kit (Qiagen). Reactions were performed with 2 μl cDNA, 2.5 μl of each primer, 2.5 μl 10× miScript Universal Primer and 10 μl 2× SYBR PCR Master Mix with the following amplification conditions: for 15 min at 95°C and for 15 s at 95°C for 40 cycles and for 60 s at 60°C. All measurements were performed using three biological replicates.

### 2.7 Data analysis

Applied Biosystems SDS software 2.3 and RQ Manager 1.2 were used to determine the Ct value. Five statistical models, including geNorm, BestKeeper, Normfinder, RefFinder and the comparative ΔCt, were used to compare gene stability. The Ct values were changed into relative quantities by using 2^–ΔCt^ (ΔCt = the corresponding Ct value − minimum Ct value). The NormFinder and GeNorm calculations depended upon these converted values; Best‐Keeper and RefFinder were used to analyse the raw Ct values. The relative expression level of the target gene was calculated according to the 2^–ΔΔCt^ method while normalising with each potential reference gene. An independent‐sample *t* test with SPSS statistical 21.0 software was employed to assess the statistical significance of the differences in gene expression. *p*‐values ≤ 0.05 were considered statistically significant.

## 3 RESULTS

### 3.1 Evaluation of the MCAO model

**FIGURE 1:**
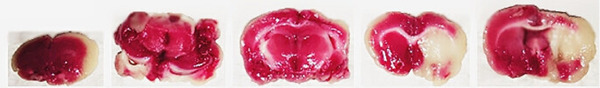
2,3,5‐Triphenyl tetrazolium chloride staining of rat brain tissue in the established model. In the middle cerebral artery occlusion (MCAO) rat, the infarcted brain area exhibited a white colour, while the non‐infarcted brain area exhibited a red colour, which demonstrated the successful establishment of the MCAO model.

The successful MCAO model showed obvious neurological deficit symptoms, and the neurological score of the established model increased significantly. Stable models with scores of 2 or 3 were selected in this study. TTC staining was also adopted to evaluate the infarction (Figure 1).

### 3.2 Specificity and amplification efficiency of reference genes

The total RNA of candidate reference genes was transcribed reversely to cDNA, which was used as a template for RT‐qPCR reaction. In these reactions, the melt curve of all candidate reference genes had a single peak, indicating that the designed primers could amplify specifically without the formation of primer dimers. The linear correlation coefficients (*R* ^2^) of all standard curves obtained by two‐fold dilution series covering a 5 log dynamic range ranged from 0.985 to 0.998. The PCR amplification efficiencies ranged from 94.8% to 113.3% (Table 1). In general, strong correlation and high efficiency were noted in all tested primers. No amplification was noted in the absence of templates.

**TABLE 1:** Amplification efficiency and correlation coefficient of candidate reference genes

Gene	Gene description	Gene ID	Primer sequences	Length (bp)	Polymerase chain reaction amplification efficiency	Linear correlation coefficient
*U6 snRNA*	*U6 small nuclear RNA*	NR_004394	5′–ATTGGAACGATACAGAGAAGATT ‐3′	106	110.9%	0.993
*5S rRNA*	*5S ribosomal RNA*	XR_004386381	5′–TCTGATCTCGGAAGCTAAGC ‐3′	119	94.8%	0.998
*miR‐24*	*miRNA 24‐1*	NR_031827	5′–GTGCCTACTGAGCTGATAT ‐3′	68	113.3%	0.987
*miR‐122*	*miRNA 122*	NR_031864	5′–TGGAGTGTGACAATGGTGTTTG ‐3′	85	101.5%	0.997
*miR‐9a*	*miRNA 9*	NR_031811	5′–TCTTTGGTTATCTAGCTGTATGA ‐3′	89	97.4%	0.985

Abbreviations: *miR‐24*, *9a* and *122*, *microRNAs‐24*, *9a* and *122*.

### 3.3 Expression of candidate reference genes in premodelling rats

**FIGURE 2:**
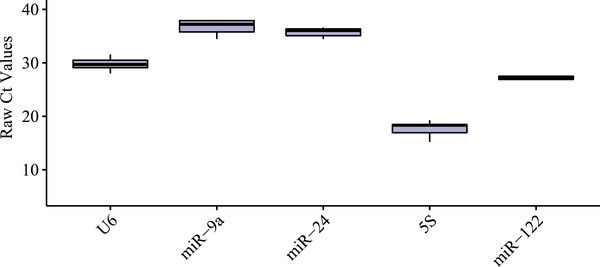
Threshold cycle (Ct) values of all candidate reference genes in all samples (*n* = 6). Boxplot of each reference gene for microRNA (miRNA) normalisation in all plasma samples of pre‐modelling rats. Each boxplot extends from the 25th to 75th percentile, with the middle line representing the median

The abundance of five candidate reference genes was detected with RT‐qPCR and presented as Ct values (Figure 2). The mean Ct values of the reference genes before modelling ranged from 17.69 (*5S rRNA*) to 36.721 (*miR‐9a‐5p*). *miR‐24* showed the least variation (with a coefficient of variation (CV) of 2.5%), whereas *5S rRNA* (with a CV of 8.6%) was the most variable reference gene. The CV of the other reference genes ranged from 4.0% to 5.6%.

### 3.4 Effects of MCAO modelling on the expression of candidate reference genes

**FIGURE 3:**
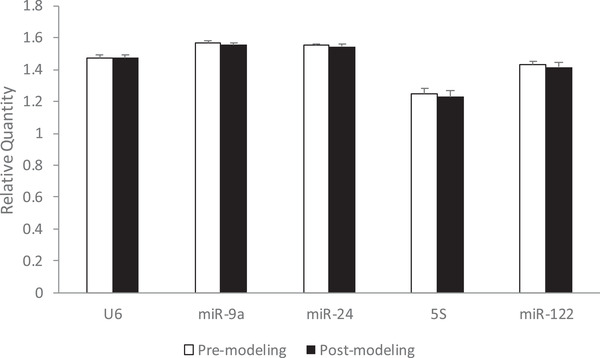
Effects of MCAO modelling on the expression of candidate reference genes (*n* = 6). The relative expression of candidate reference genes in pre‐modelling and post‐modelling rats are shown. The bars show the mean value of the relative quantity of reference genes, and the standard deviations (SDs) of the six animals are also shown. Independent‐samples *t* test between the pre‐modelling and post‐modelling rats was conducted, and no statistically significant changes were noted (*p* > 0.05).

After log‐transformation, the relative quantity of transcripts of the candidate reference genes was statistically analysed with the independent‐samples *t* test between the pre‐modelling and post‐modelling rats (Figure 3). No statistically significant changes were noted in any reference genes between the pre‐modelling and post‐modelling rats (*P* > 0.05). Therefore, MCAO modelling did not affect the expression of the candidate reference genes in this study.

### 3.5 Analysis of candidate reference genes based on the GeNorm algorithm

**FIGURE 4:**
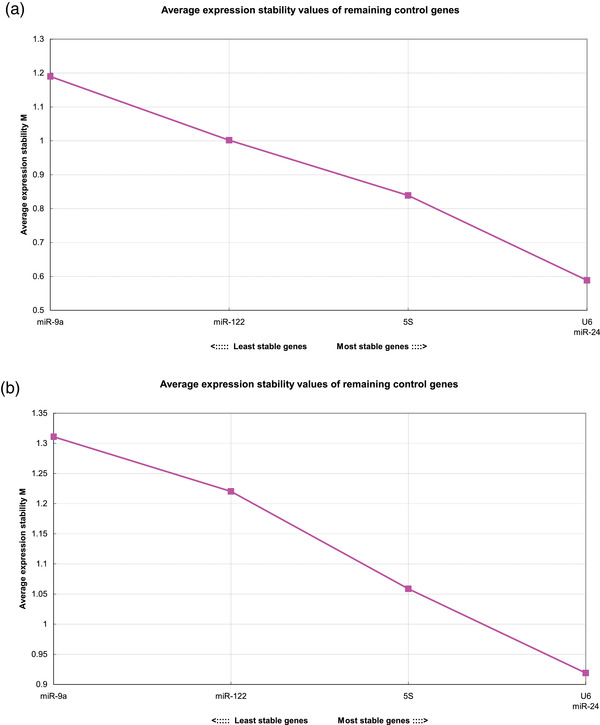
Gene expression stability ranking of candidate reference genes in pre‐modelling animals (a) and post‐modelling animals (b) based on the GeNorm algorithm. The candidate reference gene with the smallest M value was considered the most stable gene.

GeNorm analysis ranked the candidate reference genes according to their expression stability values (*M* values). The candidate reference gene with the smallest *M* value was considered the most stable gene. The high *M* values indicated high variability of gene expression. The candidate reference genes with *M* values lower than 1.5 were more reliable as stable reference genes. In our study, the *M* values of candidate reference genes ranged from 0.942 to 1.472 in pre‐modelling animals and from 1.080 to 1.447 in post‐modelling animals (Figure 4). The most stable reference gene was *miR‐24*, followed by *U6*, *miR‐122*, *5S* and *miR‐9a* in pre‐modelling animals (Table 2A), and followed by *5S*, *U6*, *miR‐122* and *miR‐9a* in post‐modelling animals (Table 2B).

**TABLE 2A:** Expression stability of candidate reference genes in pre‐modelling animals

Rank^a^	Gene Symbol	geNorm (M)	BestKeeper (SD [± CP])	NormFinder (stability value)	ΔCt (Mean SD)	Comprehensive (RefFinder)
1	*miR‐24*	0.942	0.73	0.204	1.15	*1.00*
2	*U6*	1.086	0.94	0.454	1.42	*1.68*
3	*miR‐122*	1.204	0.94	0.571	1.33	*3.22*
4	*5S*	1.245	1.23	0.698	1.59	*3.94*
5	*miR‐9a*	1.472	1.18	0.907	2.28	*4.73*

**TABLE 2B:** Expression stability of candidate reference genes in post‐modelling animals

Rank^a^	Gene symbol	geNorm (M)	BestKeeper (SD [± CP])	NormFinder (stability value)	ΔCt (mean SD)	Comprehensive (RefFinder)
1	*miR‐24*	1.080	0.70	0.207	1.25	*1.00*
2	*U6*	1.345	0.96	0.712	1.62	*2.28*
3	*5S*	1.300	1.17	0.636	1.66	*2.78*
4	*miR‐9a*	1.447	0.96	0.800	2.03	*3.98*
5	*miR‐122*	1.383	1.12	0.742	1.50	*4.00*

^a^ The listed microRNAs (miRNAs) were ranked according to the comprehensive gene stability value analysed by RefFinder. The geo mean of ranking is shown with italic numbers, which represents a recommended final ranking.

Abbreviations: CP, crossing point; Ct, threshold cycle; *miR‐24*, *9a* and *122*, *microRNAs‐24*, *9a*, *122*; PCR, polymerase chain reaction; SD, standard deviation.

The pairwise variation values (*V_n/n+_*_1_) calculated by geNorm were used to determine the optimal number of reference genes. When *V_n/n_*_+1_ < 0.15, the optimal number of reference genes is n; when *V_n/n_*_+1_ > 0.15, the optimal number of reference genes is *n* + 1 (Vandesompele et al., 2002). In this study, all values of *V_n/n_*_+1_ in pre‐modelling animals were greater than 0.15, which suggested that three stable reference genes were optimal (Vandesompele et al., 2002; Zhang et al., 2020).

### 3.6 Analysis of candidate reference genes based on the NormFinder algorithm

The algorithm principle of NormFinder was similar to that of GeNorm. The candidate reference gene with the smallest stability value was considered the most stable gene. Following this approach, the stability values of candidate reference genes ranged from 0.204 to 0.907 in pre‐modelling animals and from 0.207 to 0.800 in post‐modelling animals. The most stable reference gene was *miR‐24*, followed by *U6*, *miR‐122*, *5S* and *miR‐9a* in pre‐modelling animals (Table 2A) and followed by *5S*, *U6*, *miR‐122* and *miR‐9a* in post‐modelling animals (Table 2B).

### 3.7 Analysis of candidate reference genes based on the BestKeeper algorithm

The BestKeeper algorithm ranked the candidate reference genes based on the standard deviation (SD), CV and coefficient of correlation (*r*). A candidate reference gene with a higher *r* value and lower SD and CV values was considered a stable reference gene. The candidate reference gene with SD < 1 was acceptable. In our study, *miR‐24* had the highest *r* value of 0.96 and lowest SD and CV values of 0.73 and 2.04, respectively, followed by *U6*, *miR‐122*, *5S* and *miR‐9a* in pre‐modelling animals (Tables 2A and 3A). In post‐modelling animals, *miR‐24* was also the most stable reference gene, followed by *U6*, *miR‐9a*, *miR‐122* and *5S* (Tables 2B and 3B).

**TABLE 3A:** Expression stability of candidate reference genes in pre‐modelling animals by BestKeeper

Data of candidate reference genes from BestKeeper					
Factor	*U6*	*miR‐9a*	*miR‐24*	*5S*	*miR‐122*
*N*	6	6	6	6	6
Geo mean (CP)	29.75	36.70	35.72	17.63	26.90
Ar mean (CP)	29.77	36.72	35.73	17.69	26.93
Min (CP)	28.01	34.46	34.44	15.21	24.19
Max (CP)	31.58	38.00	36.61	19.27	28.77
SD (± CP)	**0.94**	**1.18**	**0.73**	**1.23**	**0.94**
CV (% CP)	**3.15**	**3.22**	**2.04**	**6.96**	**3.48**
Min (x‐fold)	−3.33	−4.70	−2.41	−5.36	−6.53
Max (x‐fold)	3.56	2.47	1.86	3.12	3.66
SD (± x‐fold)	1.91	2.27	1.66	2.35	1.92

BestKeeper vs.	U6	miR‐9a	miR‐24	5S	miR‐122
Coeff. of corr. (*r*)	0.857	0.594	0.956	0.885	0.883
*p*‐value	0.029	0.213	0.003	0.019	0.020

**TABLE 3B:** Expression stability of candidate reference genes in pre‐modelling animals by BestKeeper

Data of candidate reference genes from BestKeeper					
Factor	U6	miR‐9a	miR‐24	5S	miR‐122
*N*	6	6	6	6	6
Geo mean (CP)	29.76	35.83	35.27	17.03	26.16
Ar mean (CP)	29.78	35.85	35.28	17.09	26.20
Min (CP)	28.18	33.84	33.81	14.58	23.67
Max (CP)	31.78	37.04	36.43	19.03	28.26
SD (± CP)	**0.96**	**0.96**	**0.70**	**1.17**	**1.12**
CV (% CP)	**3.24**	**2.69**	**1.97**	**6.83**	**4.29**
Min (x‐fold)	−2.99	−3.97	−2.75	−5.44	−5.63
Max (x‐fold)	4.07	2.32	2.24	4.00	4.29
SD (± x‐fold)	1.95	1.95	1.62	2.25	2.18

BestKeeper vs.	U6	miR‐9a	miR‐24	5S	miR‐122
Coeff. of corr. (*r*)	0.704	0.568	0.879	0.920	0.810
*p*‐value	0.119	0.240	0.021	0.009	0.051

*Note*: *N*, number of samples; geo mean (CP): the geometric mean of CP; ar mean (CP): the arithmetic mean of CP; Min (CP) and Max (CP): the extreme values of CP; SD (± CP): the standard deviation of the CP; CV (% CP): the coefficient of variance expressed as a percentage on the CP level; Min [x‐fold] and Max (x‐fold): the extreme values of expression levels expressed as an absolute x‐fold over‐ or underregulation coefficient; SD (±x‐fold): standard deviation of the absolute regulation coefficients.

### 3.8 Analysis of candidate reference genes based on the comparative ΔCt method

The ΔCt method compared ‘pairs of genes’ and bypasses the need to accurately quantify the input RNA (Silver et al., 2006). The candidate reference genes with lower SD values were considered stable reference genes. In our study, *miR‐24* with the lowest SD values in pre‐modelling (SD value of 1.08) and post‐modelling (SD value of 1.25) rats was considered the most stable reference gene, followed by *miR‐122* and *U6* (Table 2A,B).

### 3.9 Analysis of candidate reference genes based on the RefFinder algorithm

The expression stability analysis of candidate reference genes assessed using the above four proven statistical algorithms showed basic consistency in pre‐modelling animals. However, the statistical algorithms showed inconsistency when analysed in post‐modelling animals. To further confirm the rank, a comparative stability ranking of genes based on their Ct values was performed with RefFinder. Based on the stability value, *miR‐24* and *U6* were more stable reference genes (Tables 2A,B).

### 3.10 Validation of identified reference genes

**FIGURE 5:**
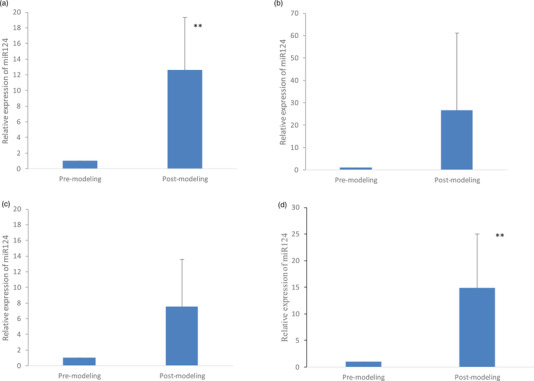
Relative expression of *miR124* in pre‐modelling and post‐modelling animals when normalised with *miR24* (a), *miR122* (b), *miR9a* (c) and the optimal combination (d). The less stable reference genes influenced the fold change and the data accuracy with a large SD

For further validation of the reliability of selected reference genes in MCAO models, the effects of the most stable reference gene *miR‐24* tested in this study, the ‘optimal combination’ of *miR‐24*, *U6* and*5S* and two less stable reference genes *miR‐9a* and *miR122* on the target gene *miR‐124* expression were investigated (Figure 5). When normalised to *miR‐24*, *miR‐124* increased by 12.60 ± 6.71‐fold (*p* ≤ 0.01) in post‐modelling animals, compared with pre‐modelling animals. When normalised with the ‘optimal combination’, *miR‐124* increased by 14.84 ± 10.2‐fold (*p* ≤ 0.01) in post‐modelling animals, compared with pre‐modelling animals. The fold changes were 7.53 ± 6.06 and 26.61 ± 34.50 when normalised to *miR‐9a* and *miR‐122*, respectively. The expression level of the target gene *miR124* was similar when the most stable reference gene *miR‐24* or the ‘optimal combination’ was used as a reference gene. However, the less stable reference genes influenced the fold change and the data accuracy with a large SD. Since we used surgical operation to establish animal models, even though the operation process was conducted in strict accordance with the method described in Section 2.2 and the evaluation criteria were consistent, there were still biological variations in individuals according to the expression level of the target gene. After normalisation, the less stable internal reference genes could amplify the variation. These results demonstrate the importance of selecting suitable reference genes for normalisation to obtain reliable results in gene expression studies.

## 4 DISCUSSION

RT‐qPCR is frequently used to study relative gene expression in molecular biological research due to its versatility and accuracy. One of the most important factors ensuring the accuracy of RT‐qPCR analyses is the stability of the reference gene selected for normalisation of gene expression data; therefore, it is crucial to select a stably and consistently expressed gene as an internal reference gene.

*miRNA‐124*, the most abundant miRNA in brain tissue, is strongly expressed in neurons, and its expression increases as neurons mature. *miRNA‐124* plays a critical role in controlling neuronal differentiation (Akerblom et al., 2012), neuroimmunity (Hamzei Taj et al., 2016), synaptic plasticity and axonal growth (Rajasethupathy et al., 2009), which exert fundamental effects on normal brain processes. Recently, accumulating studies have demonstrated that *miR‐124* is aberrant in peripheral blood and brain vascular endothelial cells following cerebral ischemia. *miR‐124* is considered to be a potential diagnostic and prognostic biomarker and pharmacological target of ischemic encephalopathy.

To date, we have not found in PubMed any report detailing the identification and validation of suitable reference genes for circulating *miR‐124* in rat MCAO modelling. In order to identify suitable reference genes, we chose two commonly used reference genes (*U6* and *5S*) and three miRNAs (*miR‐24*, *miR‐122* and *miR‐9a*) to perform RT‐qPCR. These candidate genes were chosen based on their biological functions (the gene function of *U6* is RNA splicing, and the gene function of*5S* is protein synthesis; Lardizábal et al., 2012) or their previous use as reference genes for *miR‐124* RT‐qPCR analysis (Viola et al., 2019; Vuokila et al., 2018).

In our study, we established MCAO models and calculated the variation coefficient of Ct values, which evaluated the expression dispersion of each candidate reference gene among pre‐ and post‐modelling samples. Five statistical algorithms were employed to thoroughly analyse the stability of various candidate reference genes. *miR‐24* was demonstrated to be a stably and consistently expressed gene in both pre‐modelling and post‐modelling animals that can be used as a reference control in the expression profiling analysis of *miR‐124*. Multiple reference genes are considered to be more reliable than a single reference gene for evaluating target gene expression. The optimal number of reference genes calculated by geNorm in this study was three. The ‘optimal combination’ was *miR‐24*, *U6* and *5S*. We further validated the results with *miR‐24*, the ‘optimal combination’, and two less stable reference genes, *miR‐9a* and *miR122*. The less stable reference genes led to poor normalisation and resulted in false‐negative or false‐positive results. The expression level of the target gene *miR124* was similar when the most stable reference gene *miR‐24* or the ‘optimal combination’ was used as a reference gene.

In this study, *miR24* and the optimal combination were selected as stable internal reference genes due to the stability ranking. However, different kinds or different amounts of candidate reference genes could result in different reference gene stability rankings. For example, according to Chapman and others, *GAPDH*, *ACTB* and *18S rRNA* were used as single reference genes in 72% of the summarised studies. However, the above three genes were less likely to be selected as reference genes when more candidate reference genes were included for screening. Even though *miR‐24* was demonstrated to be a stable reference gene in the MCAO model, it could not be used across all brain damage experiments. The stability of reference genes could be affected by the types and regions of the samples. In the pentylenetetrazole model of acute seizures, it was found that reference gene stability rankings varied across brain regions, including different areas of the neocortex and the dorsal vs. ventral hippocampus (Schwarz et al., 2020). To date, *GAPDH* and *β‐actin* reference genes have predominantly been used as internal reference controls due to their high and constant expression levels in many different cells and tissues (Eisenberg & Levanon, 2013; Zhu et al., 2008). However, cancerous tissues often exhibit a higher level of gene expression variability than normal tissues due to tumour heterogeneity, genetic instability and the fact that genetic alterations in diverse cancer types may differentially affect cellular processes at the transcriptome level. Therefore, Jo et al. 2019 proposed three potential reference genes (*HNRNPL*, *PCBP1* and *RER1*) to be the most stably expressed genes across various cancerous and normal human tissues (Jo et al., 2019).

In conclusion, to the best of our knowledge, this is the first study planned to screen a set of stable reference genes for *miR‐124* in rat MCAO models. For statistical analysis, the GeNorm, NormFinder, BestKeeper, RefFinder and comparative ΔCt methods were used. The above algorithms yielded the same stable gene, *miR‐24*. The ranking results of the other candidate reference genes were different, indicating the importance of using more than one software type to achieve the best result. The ‘optimal combinations’ calculated by geNorm were *miR‐24*, *U6* and *5S*. The present study is crucial for successful biomarker discovery and validation for the diagnosis of brain ischemia. Cerebral ischemia is one of the leading causes of morbidity and mortality worldwide and can trigger a vast array of pathological processes, including apoptosis, inflammation, excitotoxicity, oxidative stress and mitochondrial dysfunction, that lead to neuronal cell death (Khoshnam et al., 2017; Rodrigo et al., 2013). Therefore, we infer that *miR124* could be a good biomarker for the above pathological processes of brain damage with *miR24* and the ‘optimal combination’ of *miR‐24*, *U6* and *5S* to be stable reference genes.

## CONFLICT OF INTEREST

All authors have no conflicts of interest.

## FUNDING INFORMATION

The authors received no funding for this work.

## ETHICS STATEMENT

All applicable international, national and institutional guidelines for the care and use of animals were followed. The protocol for animal care and use was approved by the Institutional Animal Care and Use Committee of Shanghai InnoStar Bio‐Tech Co. Ltd.

## AUTHOR CONTRIBUTIONS

Jing Ma, Xijie Wang and Hui Zhou contributed to the conception of the study; Xin Yang, Jiayi Yu, Jingyi Xu, Ruiwen Zhang and Ting Zhang performed the experiments; Hui Zhou contributed significantly to the analysis and manuscript preparation.

### PEER REVIEW

The peer review history for this article is available at https://publons.com/publon/10.1002/vms3.879.

## Data Availability

The datasets used and/or analysed during the current study are available from the corresponding author on reasonable request.
